# Efficacy of phage vB_Ps_ZCPS13 in controlling Pan-drug-resistant *Pseudomonas aeruginosa* from urinary tract infections (UTIs) and eradicating biofilms from urinary catheters

**DOI:** 10.1186/s12985-025-02848-x

**Published:** 2025-07-12

**Authors:** Amira A. Mohamed, Emad M. El-Zayat, Ayman El-Shibiny

**Affiliations:** 1 Center for Microbiology and Phage Therapy, Zewail City of Science and Technology, Giza, 12578 Egypt; 2 Department of Biotechnology, Faculty of Science, Cairo University, Giza, Egypt; 3 Faculty of Environmental Agricultural Sciences, Arish University, Arish, 45511 Egypt

**Keywords:** *Pseudomonas aeruginosa*, Pan-drug resistance (PDR), Bacteriophage, Phage therapy, *Myovirus*, Biofilm, Catheters, Urinary tract infections (UTIs), Catheter-associated urinary tract infections (CAUTIs)

## Abstract

**Background:**

Pan-drug resistance (PDR) is a ticking time bomb, as it causes high human hospitalizations and mortality rates. For instance, *Pseudomonas aeruginosa* is associated with significant rates of urinary tract infections (UTIs) due to several reasons including antibiotic resistance, biofilm formation and the presence of various virulence factors. Consequently, there is an urgent need for safe and effective alternative antibacterials. Phage therapy is a promising alternative that uses naturally occurring bacteriophages (phages). Therefore, our present study investigated the isolation and characterization of a novel virulent phage (vB_Ps_ZCPS13) against the PDR *Pseudomonas aeruginosa* strain (Ps13).

**Methods:**

Phage vB_Ps_ZCPS13 was isolated from raw sewage water in Egypt during the springtime. The isolated phage was purified and amplified, followed by estimating its purity and genome size using pulsed-field gel electrophoresis (PFGE), morphology using transmission electron microscopy (TEM), antibacterial activity against other *P. aeruginosa* hosts, physiochemical stability studies, whole genome sequencing, antibiofilm activity on urinary catheters using scanning electron microscopy (SEM), and cytotoxicity assays against normal human skin fibroblast (HSF) cell lines.

**Results:**

Based on vB_Ps_ZCPS13 morphology under TEM, the phage has been classified as a myovirus. In consistent with the PFGE results, DNA sequencing revealed a phage genome size of 92,443 bp, with lytic-associated genes and no antimicrobial resistance or virulence factors. Phage vB_Ps_ZCPS13 presented a wide host range of over 93% of tested clinical isolates having different multiple antibiotic resistance (MAR) indices. Furthermore, phage vB_Ps_ZCPS13 exhibited high efficiency in plaque formation (EOP ≥ 1) against 13% of the strains and exhibited low frequencies of bacteriophage insensitive mutants (BIM). The physical stability test against harsh environmental conditions revealed phage stability within a pH range of 3.0–11.0 and stable at temperatures below 70 °C. Phage vB_Ps_ZCPS13 also exposed a significant antibacterial activity in vitro across different MOIs, with the highest reduction in bacterial growth observed at lower MOIs. Furthermore, vB_Ps_ZCPS13 demonstrated potent biofilm inhibition and clearance capabilities, effectively eradicating *P. aeruginosa* from the urinary catheter surface. Moreover, the phage presented no cytotoxicity against normal human skin fibroblast (HSF) cell lines at high titer.

**Conclusions:**

Our study offers an effective phage as a therapeutic candidate against PDR Gram-negative *P. aeruginosa* infections, including catheter-associated urinary tract infections.

## Background

The rise of antibiotic-resistant bacteria presents a significant challenge to public health on a global scale. A 2019 report from the World Health Organization (WHO) indicated that antimicrobial resistance accounts for 1.27 million deaths globally, with around 80% of these deaths associated with six bacterial species, including *Pseudomonas aeruginosa* (*P. aeruginosa*) [1, 2]. This Gram-negative bacillus is a major opportunistic pathogen, playing a key role in nosocomial, acute, and chronic infections [3]. It survives on water, diverse surfaces, and medical devices by effective binding mechanisms such as flagella, pili, and biofilms [4]. Biofilms exhibit a resistance to antibiotics that is roughly 10 to 1000 times greater than that of planktonic cells due to the limited penetration of antibiotics into the complex polysaccharide matrix (glycocalyx) of biofilms [5, 6].

As a result of the presence of biofilms and the natural and acquired mechanisms of antibiotic resistance in *P. aeruginosa*, there has been an increase in multidrug-resistant (MDR) and pan-drug-resistant (PDR) strains in recent years, with almost no fully effective antibiotics available for combating this bacterium [7]. *P. aeruginosa* has significant inherent resistance to several antibiotics, such as beta-lactams, fluoroquinolones, and aminoglycosides, resulting in high morbidity and mortality rates [8, 9]. A U.S. Centers for Disease Control and Prevention (CDC) report estimated that around 51,000 healthcare-associated infections caused by *P. aeruginosa* occur annually in the United States. Among these infections, 13% are classified as MDR, leading to approximately 400 deaths each year linked to these infections [10, 11].

Urinary tract infections (UTIs) are among the most common bacterial infections globally, affecting approximately 150 million people annually. UTIs can lead to substantial morbidity and healthcare costs, particularly as 30–50% of patients experience recurrent infections within a few months [12]. Catheter-associated UTIs pose significant challenges in healthcare settings [13], as they are the most prevalent type of healthcare-associated infection. *P. aeruginosa* leads to catheter-associated urinary tract infections (CAUTIs) by forming biofilms on the surfaces of indwelling catheters. The management of UTIs and associated complications is challenged by antimicrobial resistance (AMR) [14], underscoring the urgent need for new, well-tolerated alternatives to treat UTIs and reduce AMR.

In this alarming context, alternatives to traditional antibiotics such as phage therapy are gaining attention. Bacteriophages, often referred to as phages, are viruses that specifically target and eliminate bacteria. These entities are prevalent in various environments, particularly oceans, soil, and sewage. The application of phages for treating bacterial infections, known as phage therapy, was first introduced by d’Hérelle in 1919 [15]. This approach presents a promising alternative to antibiotics, utilizing naturally occurring phages that are recognized as part of the human microbiome [16] and exhibit a high degree of specificity for their bacterial hosts, including those that form biofilms and are resistant to antibiotics [17]. The high specificity of phages presents a therapeutic dilemma between personalized treatments and broader phage cocktails [18]. A 2022 systematic literature review of phage therapy in difficult-to-treat infections in humans revealed high tolerability and a favorable safety profile across studies reporting on various routes of administration [19]. Numerous studies have emphasized that phages can effectively eliminate *P. aeruginosa* infections and biofilm-forming bacterial cells by disrupting the biofilm’s extracellular matrix and inhibiting its formation through inhibition of quorum-sensing activity [17, 20, 21].

Phage therapy has transitioned from a hypothetical idea to a tangible, life-saving treatment alternative for individuals struggling with severe bacterial infections. In intensive care units, where managing persistent bacterial infections is critical to life-saving interventions, phage therapy is becoming an essential tool. Therefore, this research study aims to isolate and characterize virulent phage against PDR *P. aeruginosa* clinical isolates from Egyptian patient and study its therapeutic potential in resolving urinary tract infections (UTIs), including catheter-associated urinary tract infections (CAUTIs).

## Methods

### Bacterial characterization

#### Identification and growth conditions of bacterial isolates

In 2023, twenty clinical isolates of *P. aeruginosa*-infected UTI patients were gifted from the Rofayda Hospital, Giza, Egypt. The isolates were initially streaked onto selective media (Cetrimide agar, Oxoid, England) and identified based on their characteristic morphology. The identity of the bacterial strains was further verified and confirmed by the Vitek MS automated system (bioMérieux, Marcy l’Étoile, France). The bacterial colonies were then selected and stored in Tryptic Soy Broth (TSB, Oxoid, England) supplemented with 20% (v/v) glycerol at − 80 °C.

### Antimicrobial susceptibility testing

The antibiotic profiles of twenty *P. aeruginosa* isolates were evaluated against ten different antibiotics using the disc diffusion method [22]. The tested antibiotics included piperacillin (PRL; 100 µg), piperacillin-tazobactam (TZP; 110 µg), ticarcillin-clavulanate (TCC; 75/10 µg), ceftazidime (CAZ; 30 µg), cefepime (Feb; 30 µg), imipenem (IPM; 10 µg), gentamicin (CN; 10 µg), tobramycin (NN10; 10 µg), amikacin (AK; 30 µg), and ciprofloxacin (CIP; 5 µg) (Oxoid, UK). The antimicrobial susceptibility of each isolate was assessed by measuring clear zone diameters in triplicate and then calculating the mean values. The results were interpreted according to the clinical and laboratory standards institute (CLSI) 2023 [23]. The reference strain *Pseudomonas aeruginosa* NCTC 12,903 / ATCC 27,853 was used as a quality control in the susceptibility test. Additionally, the multiple antibiotic resistance (MAR) index for each isolate was determined based on the method described by [24].

### Phage isolation, purification, and amplification

Different liquid sewage samples were collected from October Gardens Water Station, Giza, Egypt. The sewage samples were subsequently centrifuged at 8000 rpm for 10 min, then the phage isolation was carried out through enrichment technique. Each collected sewage sample was mixed with an equal volume of a mixed culture of *P. aeruginosa* isolates in a sterile 50 mL centrifuge tube as previously described [25, 26]. The mixtures were incubated in the shaking incubator for 4 h at 37 °C. After incubation, the samples were centrifuged at 8000 rpm for 10 min. The supernatant was then filtered through a 0.22 μm membrane filter to remove bacterial cells, and 1% chloroform was added.

Following the double agar method described by Clokie & Kropinski, with a slight modifications [27], 80 µL of exponential-phase bacterial host culture was mixed with 5 mL of soft agar (0.5% w/v) maintained at 45–50 °C. The mixture was poured onto a TSA plate, after that 10 µL was spotted onto the bacterial lawns in triplicate, and the plates were incubated at 37 °C for 24 h. Then, single plaques observed on the plates were picked using sterile pipette tips, suspended in 100 µL of sterilized Gelatin-SM buffer [5.8 g NaCl, 2.0 g MgSO₄·7 H₂O, 50 mL 1 M Tris-HCl (pH 7.4), and 1 L dH₂O] and stored at 4 °C for 4 h.

Purification of phages was achieved through a tenfold serial dilution of each single plaque, followed by repeated spot assays (six rounds) to obtain a single phage. To amplify phage concentration, 10 mL of bacterial host culture was infected with 100 µL of a single phage and incubated at 37 °C for 4 h, followed by centrifugation at 8000 rpm for 10 min and transferring the supernatant to a new centrifuge tube. A spot assay was then conducted as previously described to quantify the plaque-forming units (PFU)/mL.

### Phage characterization

#### Pulsed-field gel electrophoresis (PFGE)

PFGE analysis was performed to estimate the purity and the genome size of the isolated phage (vB_Ps_ZCPS13) following the method described by Lingohr et al., with slight modifications [28]. In summary, 100 µL of phage suspension (10⁹ PFU/mL) was added to 100 µL of 1.4% plug agarose in plugs mold. After solidification, the plugs were immersed in lysis buffer containing 1 mg/mL proteinase K (ThermoFisher Scientific, USA), 0.2% w/v SDS (Sigma Aldrich, UK), 100 mM EDTA, and 1% w/v N-Lauryl sarcosine (Sigma Aldrich, UK), and the mixture was incubated at 55 °C for 18 h.

After the plugs were washed and loaded onto a PFGE gel composed of 1.5% agarose along with a Lambda PFG Ladder (Biolabs, UK) as a size standard, electrophoresis was carried out using a Bio-Rad CHEF DRII system (Bio-Rad, USA) for 18 h at 200 V (6 V/cm), with a switch time ranging from 30 to 60 s. The gel was then stained with 5 µL of ethidium bromide (Carl Roth, Germany) for visualization, and washed in distilled water, then analyzed under UV light using the ChemiDoc imaging system (Bio-Rad, USA).

### Morphological characterization by transmission electron microscopy (TEM)

Phage vB_Ps_ZCPS13 was visualized using a JEOL 1230 transmission electron microscope (TEM) at the Faculty of Science, Alexandria University, Egypt. The phage suspension (10⁹ PFU/mL) was prepared in SM buffer, filtered, and applied to Formvar carbon-coated copper grids (Pelco International). The grids were stained with 2% phosphotungstic acid (pH 7.0) and dried before TEM examination.

### Host range analysis

The lytic activity of the isolated phage was evaluated against a total of 30 clinical isolates of *P. aeruginosa* using the spot assay, which was performed in triplicate as previously described [29]. This included 20 primary isolates obtained from Rofayda Hospital, Giza, Egypt, which were fully characterized in this study, and an additional 10 clinical isolates to extend the host range assessment.

Briefly, to evaluate each bacterial isolate, 80 µL of fresh culture was added to 5 mL of soft agar (0.5% w/v) and poured onto a TSA plate. Afterwards,10 µL of phage lysate was spotted onto the bacterial lawns, and the plates were incubated at 37 °C for 24 h.

### Relative efficiency of plating (EOP)

The effectiveness of the phage against *P. aeruginosa* host strains was further evaluated using the efficiency of plating (EOP) method as previously described [29]. In this method, the phage stock was serially diluted 10-fold, ranging from 10¹ to 10⁸, and 10 µL of each dilution was spotted in triplicate onto a fresh layer of each susceptible bacterial isolate identified through the host range assay. Plaques with the highest phage titer were counted after overnight incubation, and the average plaque-forming unit (PFU) count for each spot was calculated in triplicate for each bacterial isolate. The EOP was calculated by dividing the average PFU count of the target bacteria by the average PFU count of the host bacteria.

### Determination of the frequency of bacteriophage insensitive mutants (BIMs)

To determine the BIM, the susceptible bacterial host Ps13 was treated with the phage at a multiplicity of infection (MOI) of 100. After 10 min of incubation at 37 °C, the suspension was serially diluted and spotted onto plates using the double agar overlay plaque assay in triplicate, then the plates were incubated overnight. BIM was calculated by dividing the number of viable bacteria remaining after phage infection by the initial viable bacterial count [30].

### Physical stability of phage vB_Ps_ZCPS13

The stability of phage vB_Ps_ZCPS13 was evaluated under various conditions, including temperature, pH, and UV exposure. The thermal stability was evaluated by incubating the phage suspended in SM buffer at temperatures of -20 °C, 4 °C, 37 °C, 50 °C, 60 °C, 70 °C, 75 °C, and 80 °C for 1 h. Following incubation, the phage was quantified via tenfold serial dilution and assessed in triplicate via the spot test assay as outlined in previous study [31].

Furthermore, the pH stability was assessed by incubating the phage suspended in deionized water, adjusted to pH values of 2, 3, 5, 7, 9, 11, and 12 with HCl and NaOH for 1 h. The phage was subsequently quantified using the spot test assay.

Additionally, the susceptibility of the phage to UV inactivation was assessed by directly exposing the phage suspended in SM buffer to UV radiation (λ = 253 nm). Samples were collected at 15-minute intervals over the course of 1 h, and phage titers were determined using a spot test assay performed in triplicate.

### In vitro experiments to study phage replication dynamics at different MOIs

The bacteriolytic activity of the phage was evaluated in vitro against the bacterial host (Ps13) at multiple multiplicities of infection (MOIs) [31]. Phage lysates with concentrations of 10^5^, 10^6^, and 10^7^ PFU/mL were prepared to achieve MOIs of 0.1, 1, and 10, respectively. The bacterial host was cultured in TSB to reach the mid-log phase, with a concentration of 10^6^ colony-forming units (CFU)/mL, confirmed by serial dilution and spot assay on agar plates. For each MOI, 20 mL of bacterial suspension was divided into two 10 mL aliquots: one for testing and the other for the control. The volume of the phage lysate was calculated based on each MOI. The test and control tubes were incubated in a shaker incubator at 37 °C and 100 rpm/min.

100 µL were taken from each tube at 15 distinct time points: 0, 10, 20, 30, 45, 60, 75, 90, 120, 150, 180, 210, 240, 270, and 300 min. The collected samples were serially diluted tenfold in TSB, and each dilution was spotted onto agar plates. The bacterial counts in the control tube were calculated as CFU/mL. The surviving bacterial counts and free phage titers were calculated as CFU/mL and PFU/mL, respectively. The bacterial survival rates at the different MOIs were analyzed to identify the MOI with the most efficient bacteriolytic activity. These data provide insight into the optimal phage-to-bacterium ratio for effective bacterial eradication [32].

### Genomic DNA extraction and sequencing

The genomic DNA of the isolated phage was extracted from 10 mL of purified high-titer phage lysate (10¹⁰ PFU/mL) using the phenol–chloroform–isoamyl alcohol method as previously described [33]. The DNA concentration and quality were then measured using the FLUOstar Omega Microplate Reader (BMG LABTECH, Germany). Nucleotide sequencing was subsequently performed on the Illumina MiSeq platform. The sequence reads were de novo assembled using Unicycler (v0.4.8) via the BV-BRC portal. The accuracy of the paired-end DNA reads was assessed through FASTQC [34].

### Bioinformatics analysis of phage vB_Ps_ZCPS13

Genome visualization, comparison, and orientation were carried out using ProgressiveMauve and Ugene, with the *Pseudomonas* phage PAK_P4 (accession number NC_022986) as a reference [35, 36]. The assembled genome was annotated using the Rapid Annotation using the Subsystem Technology Toolkit (RASTtk) [37]. Another round of annotation was conducted after RASTtk annotation to assign functions to proteins with unassigned functions. For this purpose, tools such as NCBI BLASTp, UniProt Blast, PhageScope, HHPred, and InterProScan were used. The circular genomic map was generated utilizing CGView on the PROKSEE server [38].

The identification of temperate genetic markers, bacterial virulence factors, and antimicrobial resistance genes was conducted using BACterioPHage LIfestyle Predictor (BACPHLIP) and PhageLeads [39, 40]. The topology of the phage-predicted proteome was analyzed for transmembrane domains (TMDs) using DeepTMHMM [41]. Furthermore, genes with putative depolymerase function were also detected by Phage Depolymerase Finder (PhageDPO) [42].

### Phylogenetic analysis

Circular and rectangular proteomic trees were constructed using the ViPTree server [43]. The results from ViPTree were used to identify phages exhibiting the highest tBLASTx scores (*S*_*G*_) and outgroup phages with the lowest *S*_*G*_ scores. These, along with the top BLASTn hits from NCBI were used as inputs for the virus intergenomic distance calculator (VIRIDIC). The VIRIDIC tool is used to calculate pairwise intergenomic similarities between phage vB_Ps_ZCPS13 and the selected phages [44]. To address the taxonomic status of vB_Ps_ZCPS13, all established species of *Pakpunavirus* listed in the ICTV database were included in the analysis [45]. VIRIDIC was re-run accordingly to clarify whether vB_Ps_ZCPS13 represents a novel species or a new strain within the genus *Pakpunavirus*.

Further analysis of the closest-related phages was conducted using CoreGenes 5.0 to identify genes conserved across the genus and family levels [46]. The terminase large subunit (TerL), a signature gene, was used to construct a protein-based phylogenetic tree using MEGA11 [47], employing the CLUSTAL-W aligner and the best maximum likelihood fit model.

### Screening for bacteriophage potency against bacterial biofilm

Phage antibiofilm activity was assessed at various MOIs (100, 10, 1, 0.1, 0.01, 0.001, and 0.0001) for two phenotypes: inhibition of biofilm formation and clearance of preformed biofilm. The biofilm formation, staining, and measurement were performed using the microtiter plate biofilm assay as previously described with slight modifications [48].

A fresh bacterial culture in TSB was adjusted to an exponential phase concentration of 10⁵ CFU/mL, confirmed by serial dilution and spot assay on agar plates, and 180 µl of this culture was added into the wells of polystyrene microtiter plates (Greiner Bio-One, Portugal). For controls, a row of six wells on each microtiter plate contained 200 µl of untreated culture, consisting of 180 µl of bacterial suspension and 20 µl of TSB (the same diluent used in phage MOI preparations).

### Biofilm inhibition assay

The bacterial cultures were subjected to phage treatment from the start of the experiment and incubated at 37 °C for 48 h. Each MOI was tested in six replicate wells, with MOIs ranging from 0.0001 to 100, corresponding to phage concentrations of 10² PFU/mL to 10⁸ PFU/mL. A 20 µL volume of phage, adjusted to the required MOI, was added to each well.

### Biofilm clearance assay

The biofilm clearance assay was conducted in the same approach as the inhibition assay, with the exception that bacterial cultures were first incubated in the wells for 48 h without phage treatment to allow for mature biofilm formation. Following incubation, phage preparations were introduced at various MOIs to the untreated wells and incubated at 37 °C for 24 h.

Planktonic cells and media were discarded in both assays, and the wells were washed by PBS to remove unattached cells. The plates were then inverted to remove residual liquid and air-dried. The adhered biofilm was stained with 200 µl of freshly prepared 0.1% (w/v) crystal violet. The excess stain was discarded, and the plates were rewashed and dried at room temperature. The stain was solubilized in 200 µl of 30% (v/v) acetic acid to quantify the biofilm, and the optical density of the solubilized biofilm was measured at 590 nm using the FLUOstar^®^ Omega Microplate reader (BMG LABTECH, Germany).

### Bacteriophage potency against bacterial biofilm on urinary catheter surfaces

The antibiofilm activity of the phage on urinary catheter surfaces was evaluated at the chosen MOI 1 for the two phenotypes: biofilm inhibition and biofilm clearance. Biofilm experiments were conducted in Wasserman tubes, containing silicone urinary catheters (size 14) cut into 1 cm segments, each placed in individual tubes. Fresh TSB-diluted bacterial culture was subsequently grown to a concentration of 10^5^ CFU/mL, confirmed by serial dilution and spot assay on agar plates, and phage lysate with a concentration of 10^6^ PFU/mL. For each sample, the catheter segment with untreated culture was used as a negative control. Biofilm inhibition and clearance followed the same methodology as described for the biofilm assay performed in the microtiter plate, except that the total volume used was 4 mL (3.6 mL bacterial culture and 0.4 mL phage lysate).

After incubation at 37 °C, each catheter segment was washed three times with PBS to remove planktonic cells. The catheter segments were transferred to new Wasserman tubes containing 1mL saline and followed the vortexing–sonication–vortexing (V-S-V) method to dislodge adherent cells from the catheter surface [49]. The number of viable bacterial cells was subsequently quantified by performing a spot test assay on serially diluted samples. For further qualitative analysis using a scanning electron microscopy (SEM), catheter samples were fixed with 2.5% glutaraldehyde, followed by gold sputter coating.

### Phage cytotoxicity effect on normal human cell lines

The MTT **(**3-(4,5-dimethylthiazol-2-yl)-2,5-diphenyltetrazolium bromide) test was used to determine the effect of the phage on the viability of normal human skin fibroblast (HSF) cells. The HSF cells were grown in high-glucose Dulbecco’s Modified Eagle’s Medium (DMEM, Biowest, France) which contained 10% fetal bovine serum, 100 units/mL penicillin, and 100 mg/mL streptomycin. The cells were seeded at a density of 8 × 10³ cells per well in 96-well plates (CELLSTAR, Greiner Bio-One, Portugal) and incubated at 37 °C in a carbon dioxide incubator.

A purified lysate of the phage at a concentration of 10¹⁰ PFU/mL was applied to a proliferative monolayer of fibroblasts in wells containing a total volume of 200 µL. The effect of the phage on cell growth was examined after 24 h. Untreated cells in DMEM served as a negative control, while SM buffer diluted in DMEM with untreated cells served as the vehicle control.

Following incubation, the DMEM media was replaced with 100 µL of fresh DMEM containing 10% MTT labeling reagent (final concentration of 0.5 mg/mL) and incubated for an additional 4 h. The media was then carefully removed, and 100 µL of DMSO was added to each well to dissolve the formazan crystals formed. The absorbance of the resulting solution was measured at 570 nm using a FLUOstar Omega Microplate Reader. The optical density (OD) values were used to calculate cell viability as a percentage of the untreated cells (negative control) using the following formula [50, 51].

$$\begin{aligned}&Cell\:viability\\& \quad =100-\left(\left(\:1-\frac{{OD}_{phage-treated\:cells}-{OD}_{blank}}{{OD}_{untreated\:cells}-{OD}_{blank}}\:\right) \times\:100\right)\end{aligned}$$

### Statistical analysis

All experiments were performed in triplicate, and the results were presented as the mean ± standard deviation (SD). Statistical analyses and graph generation were carried out using GraphPad Prism software. ANOVA and t-tests were used throughout the study to determine the significance values.

## Results

### Bacterial characterization

The clinical bacterial isolates exhibited fluorescent green coloration on cetrimide agar and were further identified as *P. aeruginosa* with 99.9% confidence using the Vitek MS system.

### Antimicrobial susceptibility testing

**Fig. 1:**
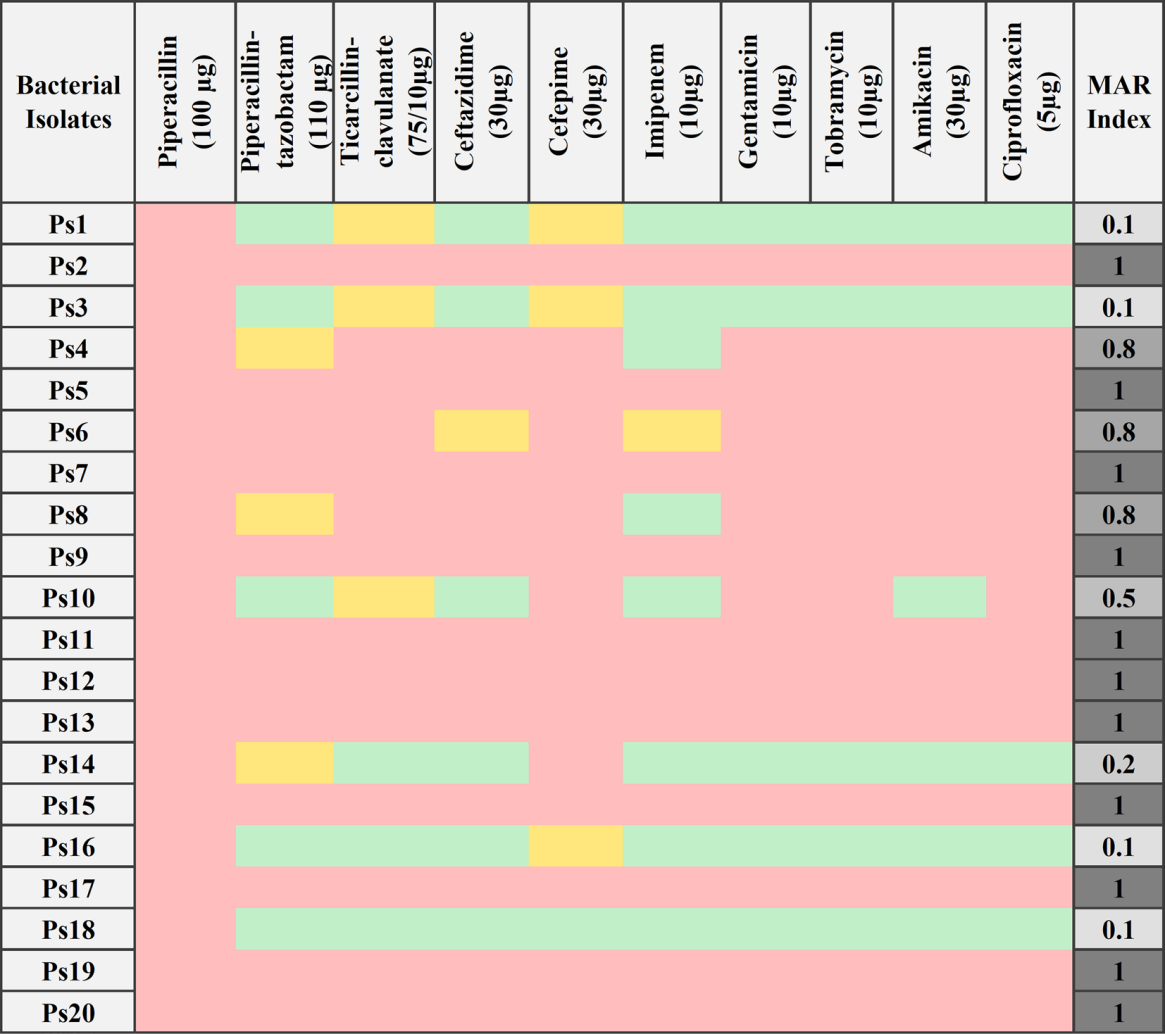
Antibiotic sensitivity profile for 20 UTI *P. aeruginosa* isolates over 10 antibiotics (sensitivity is indicated in green, intermediate in yellow, and resistance in pink) with their MAR index values

Bacterial susceptibility to antibiotics is categorized into three classifications: sensitive, intermediate, and resistant. The results revealed that *P. aeruginosa* isolates exhibited varying sensitivity responses across antibiotic classes (Fig. 1). About 55% of bacterial isolates were categorized as PDR (with MAR Index 1), and 20% were classified as MDR (15% with MAR Index 0.8 and 5% with MAR Index 0.5). The data indicated that piperacillin was ineffective against all isolates. Moreover, the isolates exhibited 80% resistance to cefepime, 75% resistance to gentamicin, tobramycin, and ciprofloxacin, 70% resistance to ticarcillin-clavulanate, and amikacin, 65% resistance to ceftazidime, 60% resistance to piperacillin-tazobactam, and 55% resistance to imipenem. A PDR representative isolate, Ps13, was selected for the study experiments.

### Morphology of phage vB_Ps_ZCPS13

**Fig. 2:**
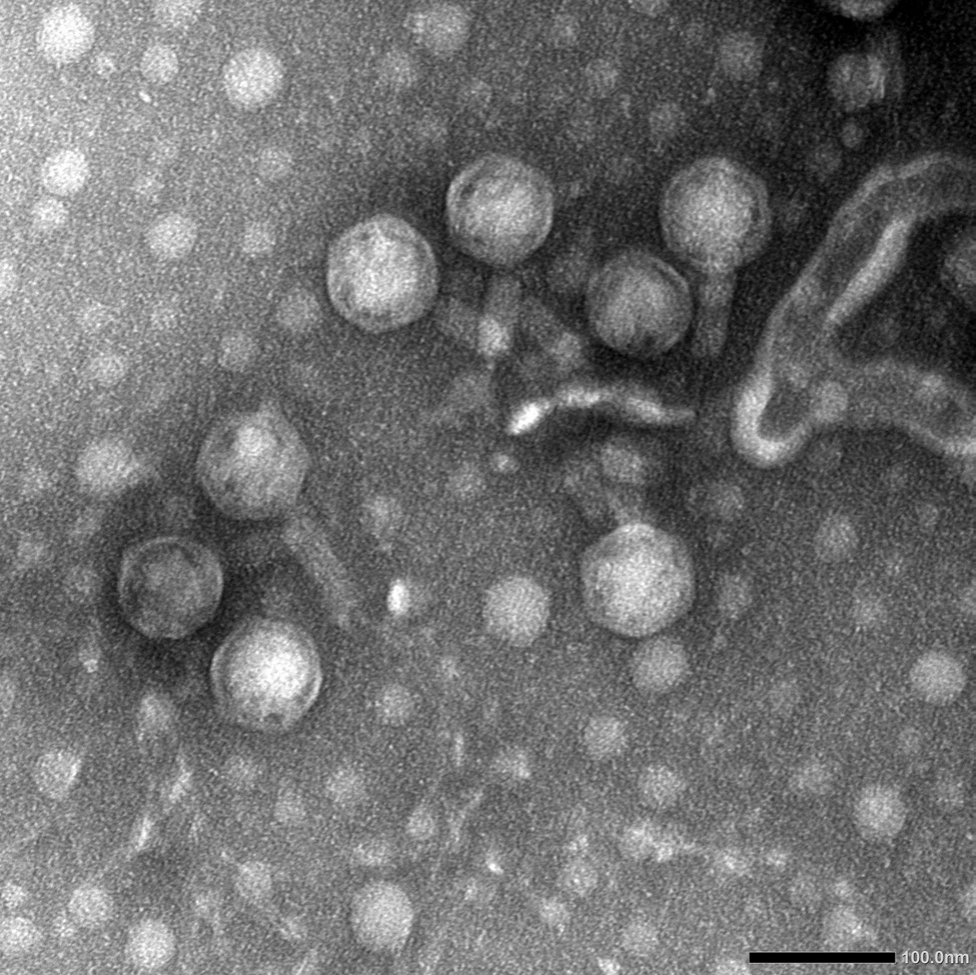
Transmission electron micrograph of phage vB_Ps_ZCPS13, scale bar; 100.0 nm

Phage vB_Ps_ZCPS13 is classified as a myovirus, featuring an icosahedral capsid and a contractile tail, as observed through TEM (Fig. 2) and consistent with the guidelines of the International Committee on Taxonomy of Viruses (ICTV). Phage vB_Ps_ZCPS13 possesses an icosahedral head with estimated dimensions of approximately ~ 78 nm in height and ~ 72 nm in width, along with a tail measuring about ~ 90 nm in length and ~ 20 nm in width. These virion measurements were obtained using ImageJ version 1.54 g.

### Host range determination and relative efficiency of plating (EOP)

**Fig. 3:**
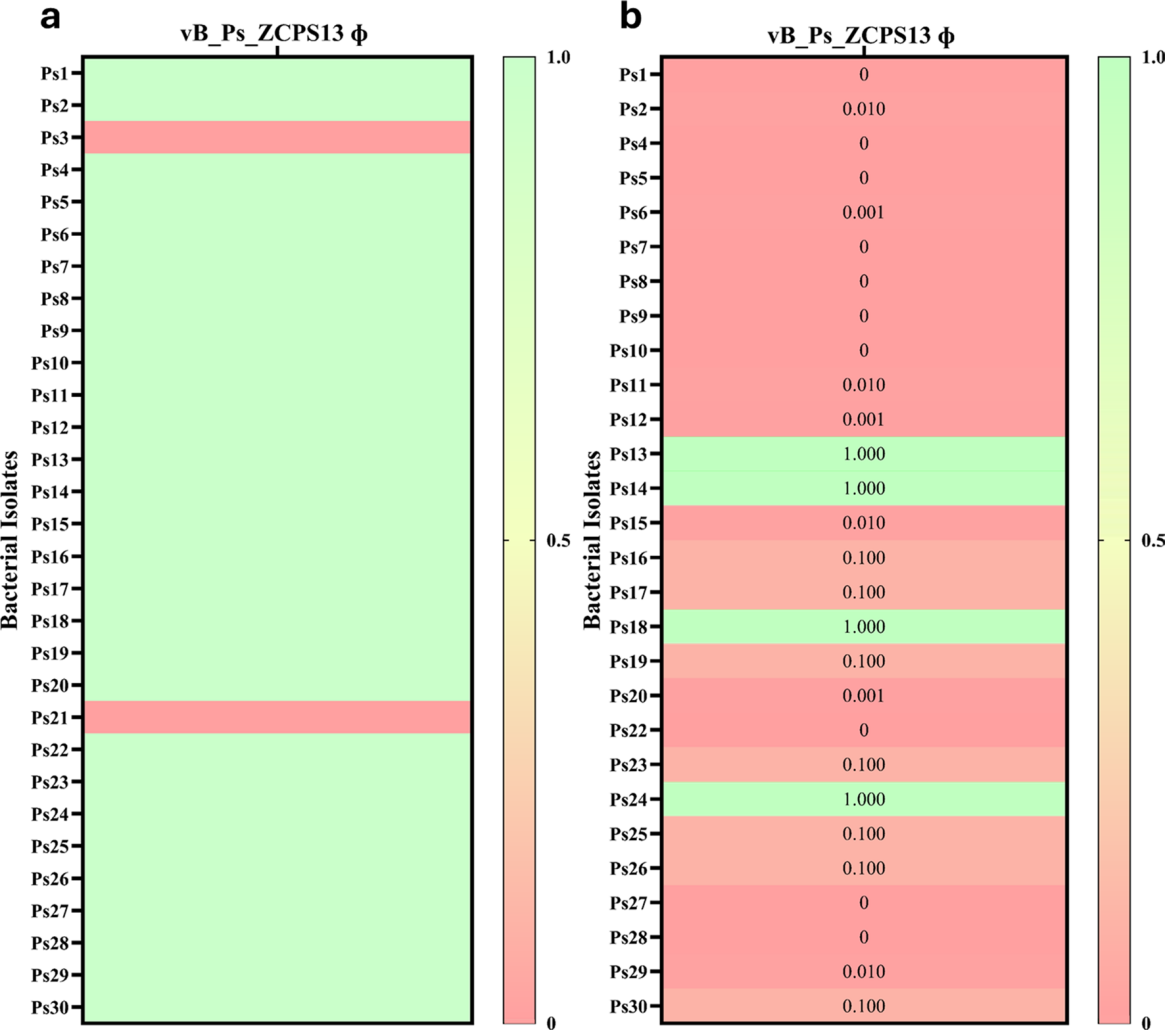
Heatmap of host range and EOP for different *P. aeruginosa* isolates tested against phage vB_Ps_ZCPS13. (**a**) The host range results represent light green for lysed bacteria and red for resistant bacteria. (**b**) The EOP values are represented as green (≥ 1), light red (≥ 0.001 and < 1), and dark red (< 0.001)

Phage vB_Ps_ZCPS13 against different resistant bacterial strains exhibited clear zones (indicative of bacterial lysis) in the spotting area. Phage vB_Ps_ZCPS13 lysed 28 out of the 30 tested *P. aeruginosa* isolates. The results indicated that phage vB_Ps_ZCPS13 demonstrated a wide host range, displaying lytic activity against 93% of the tested *P. aeruginosa* clinical isolates (Fig. 3a).

The virulence of phage vB_Ps_ZCPS13, specifically its lytic potential, was further evaluated by analyzing the relative EOP (Fig. 3b). The bacterial isolate Ps13 was the indicator host for calculating the relative EOP. Among the 28 susceptible *P. aeruginosa* isolates, 4 presented an EOP value equal to 1, whereas the remaining 24 isolates presented an EOP value < 1.

### Determination of the frequency of bacteriophage insensitive mutants (BIM)

The bacteriophage insensitive mutants (BIM) assays were conducted in vitro to evaluate the long-term effectiveness of phage vB_Ps_ZCPS13. The BIM frequency was found to be 0.001263 ± 0.000893.

### Physical stability of phage vB_Ps_ZCPS13

**Fig. 4:**
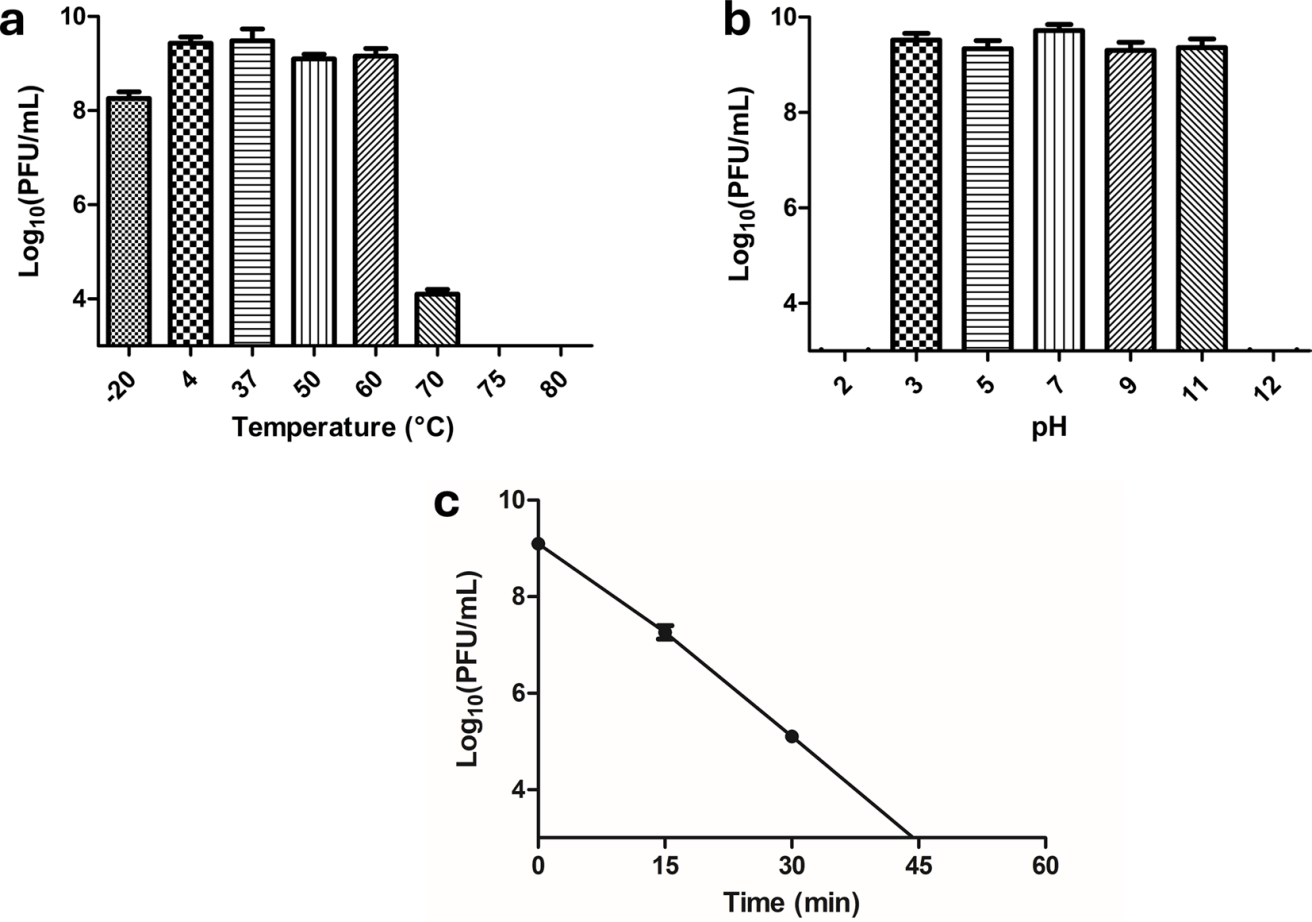
Phage vB_Ps_ZCPS13 physicochemical stability at different temperatures (**a**), pH values (**b**), and under a UV lamp (**c**)

Phage vB_Ps_ZCPS13 was stable at storage temperatures (-20 °C and 4 °C) and remained stable up to 60 °C without reduction in phage titer. While at 70 °C, the titer of the phage was reduced by 5 log_10_ PFU/mL. At higher temperatures of 75 °C and 80 °C, the titer dropped drastically below the detection limit (Fig. 4a).

Phage vB_Ps_ZCPS13 demonstrated an optimal pH stability range of 3.0–11.0, maintaining excellent activity without reduction of phage titer. However, it became inactive when exposed to extreme pH values of 2.0 and 12.0 (Fig. 4b).

Under UV light exposure, the phage titer gradually decreased over 45 min, with a 2 log_10_ PFU/mL reduction observed after 15 min, followed by an additional 2 log_10_ PFU/mL reduction at 30 min until it was completely inactive at 45 min (Fig. 4c).

### Replication dynamics of phage vB_Ps_ZCPS13

**Fig. 5:**
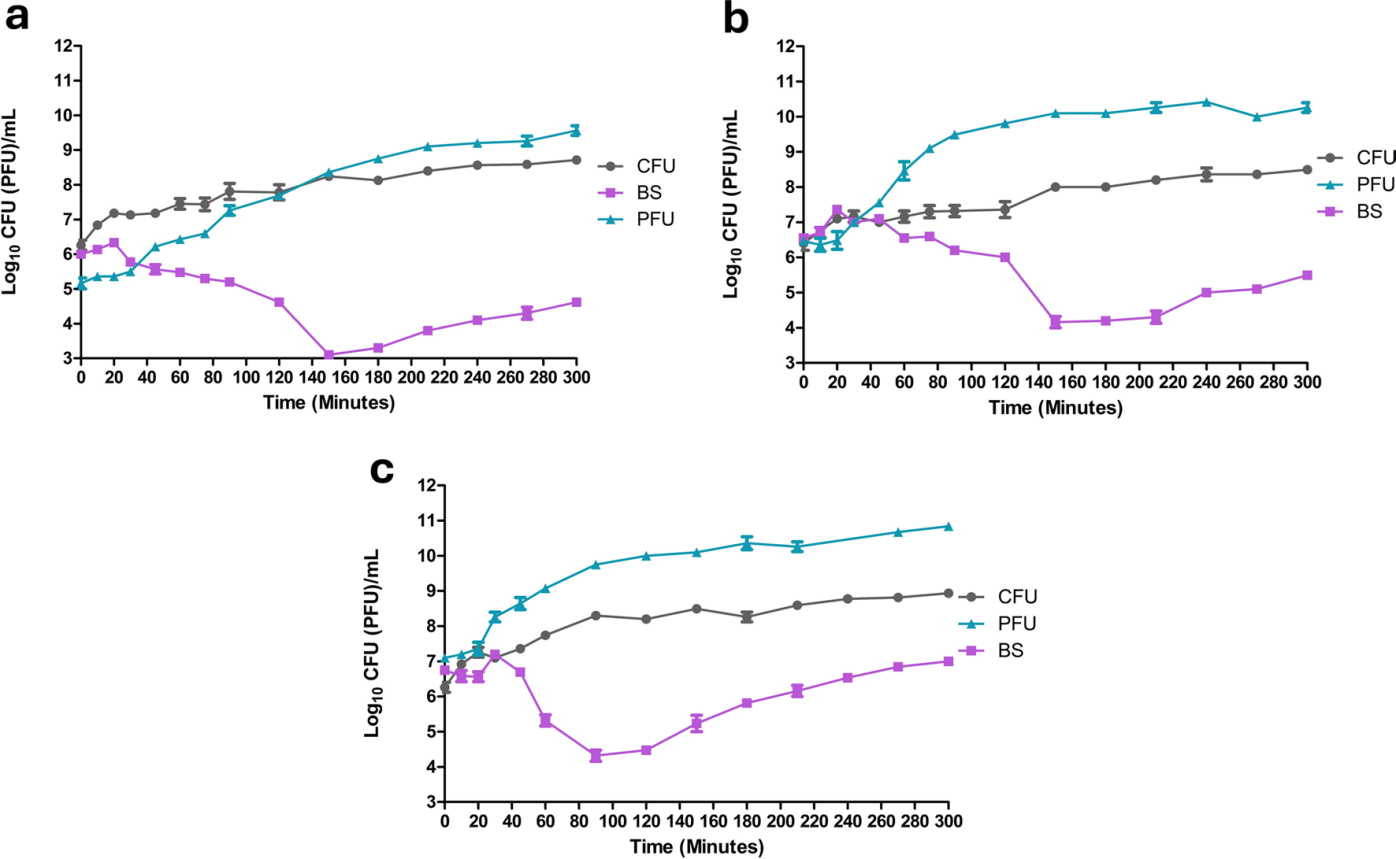
The phage-bacterial dynamics of phage vB_Ps_ZCPS13 at 37 °C. (**a**) Phage infection with MOI 0.1, (**b**) Phage infection with MOI 1, and (**c**) Phage infection with MOI 10. CFU: Colony-forming unit; PFU: Plaque-forming unit

The phage’s bacteriolytic activity was determined by the apparent reduction in bacterial host growth in the broth culture treated with the phage compared to the untreated (control) bacterial culture. The dynamics of phage antibacterial activity varies depending on the MOI used (Fig. 5). Bacterial growth was significantly reduced (*P* < 0.05) after 30 min at MOIs 0.1 and 1, whereas it required 45 min at MOI 10. Bacterial regrowth or resistant bacteria was observed at 180 min for MOIs 0.1 and 1 and at 120 min for MOI 10.

At the end of the 300 min, the bacterial count in the treated cultures was significantly reduced (*P* < 0.0001) by 5.04 log_10_, 4.77 log_10_, and 3.84 log_10_ CFU/mL for MOIs 0.1, 1, and 10, respectively, compared to the control. The production of phage vB_Ps_ZCPS13 significantly increased (*P* < 0.0001) at MOI levels of 0.1, 1, and 10, with increases of 4.41 log_10_, 3.79 log_10_, and 3.74 log_10_ PFU/mL, respectively. These findings indicate that phage vB_Ps_ZCPS13 exhibited significant antibacterial lytic activity at all tested MOIs, with the greatest reduction in bacterial growth by 5.04 log_10_ (*P* < 0.0001). and increased phage production by 4.41 log_10_ (*P* < 0.0001) occurring at the lower MOI of 0.1.

### Genome sequencing and bioinformatics analysis

**Fig. 6:**
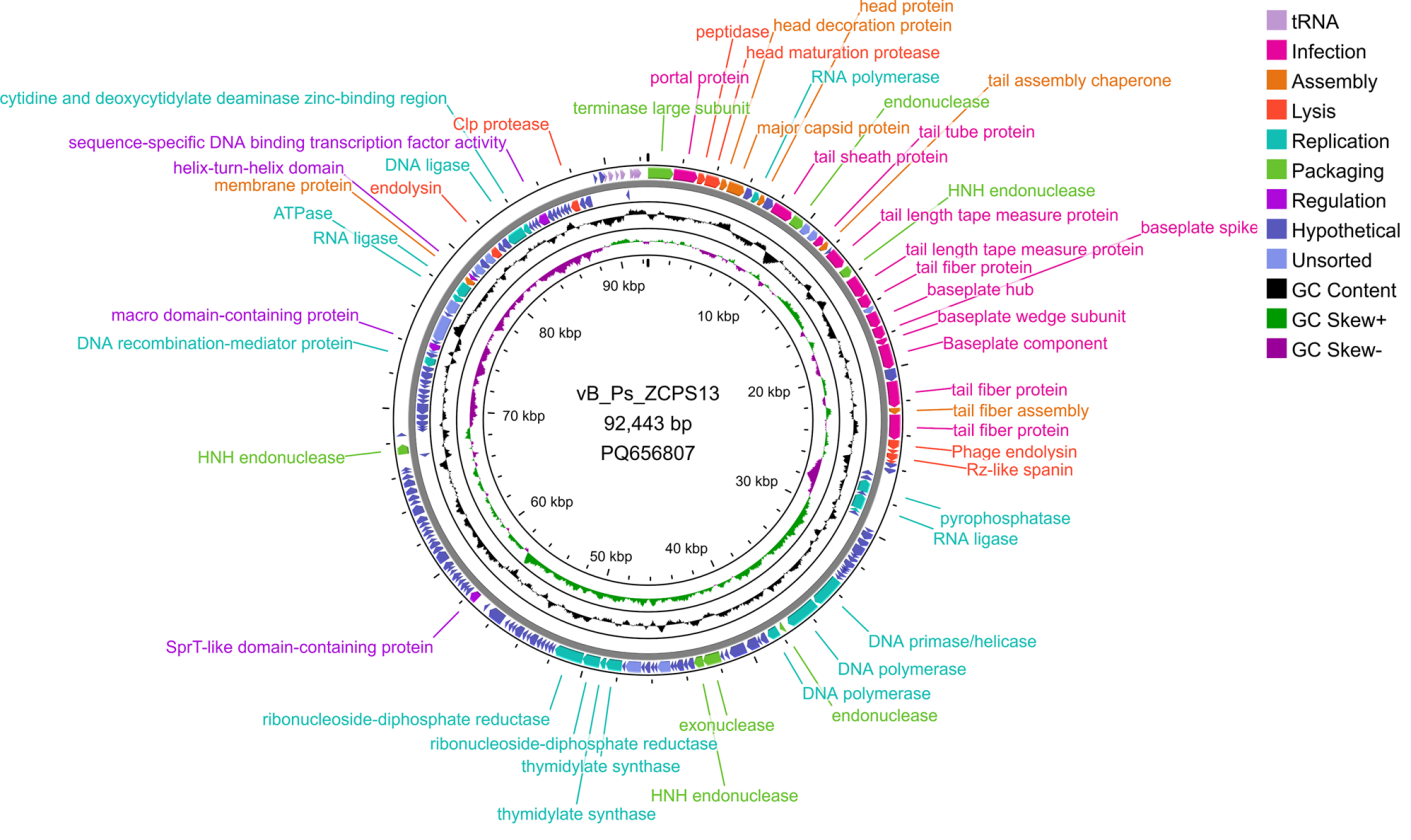
Genomic circular map of phage vB_Ps_ZCPS13. Coding sequences (CDSs) are represented in different colors according to the predicted functional category. The GC content skew is represented in the middle circle. The map was constructed by Proksee [38]

**Fig. 7:**
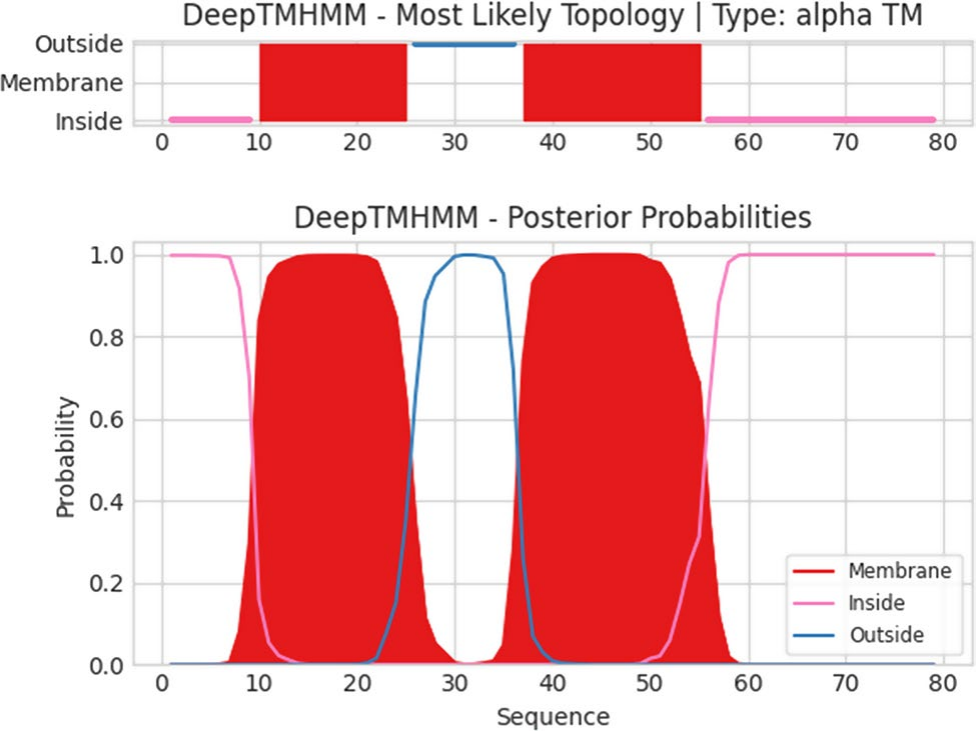
Predicted topology of ORF 32 (putative class II holins) by DeepTMHMM. The top part of the chart represents the topology of the predicted domain. The probability of the predicted topology was 100%

Consistent with the PFGE results, DNA sequencing of phage vB_Ps_ZCPS13 genome revealed one contig double-stranded DNA of 92,443 bp length and GC content of 49.24%. The annotated genome of the phage has been deposited in the NCBI GenBank under the accession number PQ656807. The predicted open reading frames (ORFs) in the complete genome of vB_Ps_ZCPS13 were 188, of which contributed to several protein functions, including DNA genome infection (12 genes), assembly (6 genes), lysis (8 genes), replication (16 genes), packaging (7 genes), and regulation proteins (4 genes) along with tRNA genes (12 genes). The genomic map highlights functional genes and their positions in the genome (Fig. 6). The PhageDPO tool identified depolymerase activity in the tail fiber protein (ORF 28 and 30) with 89.99% and 82.47% predictions, respectively. DeepTMHMM analyzed the transmembrane domains (TMDs), predicted in 12 putative proteins (ORFs: 18, 20, 32, 35, 36, 60, 64, 69, 107, 133, 134 and 150). The topology of 1 TMD was detected in a putative holin (ORF 32), including two alpha-helical transmembrane domains with 100% probability (Fig. 7). Phage vB_Ps_ZCPS13 exhibits a virulent lifestyle as predicted by BACPHLIP with 100% probability. Likewise, PhageLeads did not detect either temperate lifestyle genes or antimicrobial resistance or virulence genes, suggesting the safety and potential of phage vB_Ps_ZCPS13 for therapeutic applications.

### Phylogenetic analysis

**Fig. 8:**
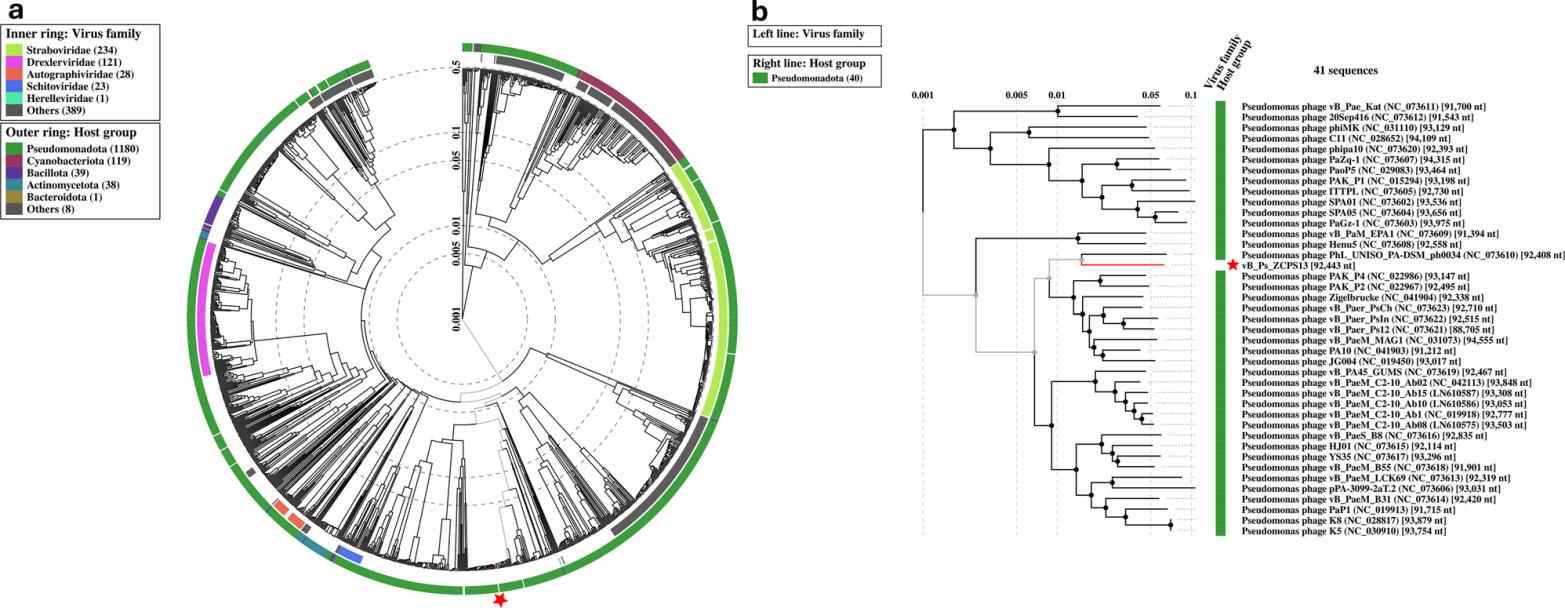
Proteomic tree of phage vB_Ps_ZCPS13. (**a**) Circular proteomic tree based on genome-wide similarities of phage vB_Ps_ZCPS13 (highlighted with a red star), along with its closely related reference phage genomes. (**b**) Rectangular proteomic tree comparing phage vB_Ps_ZCPS13 with 40 phages that have the highest ViPTree *S*_*G*_ scores

**Fig. 9:**
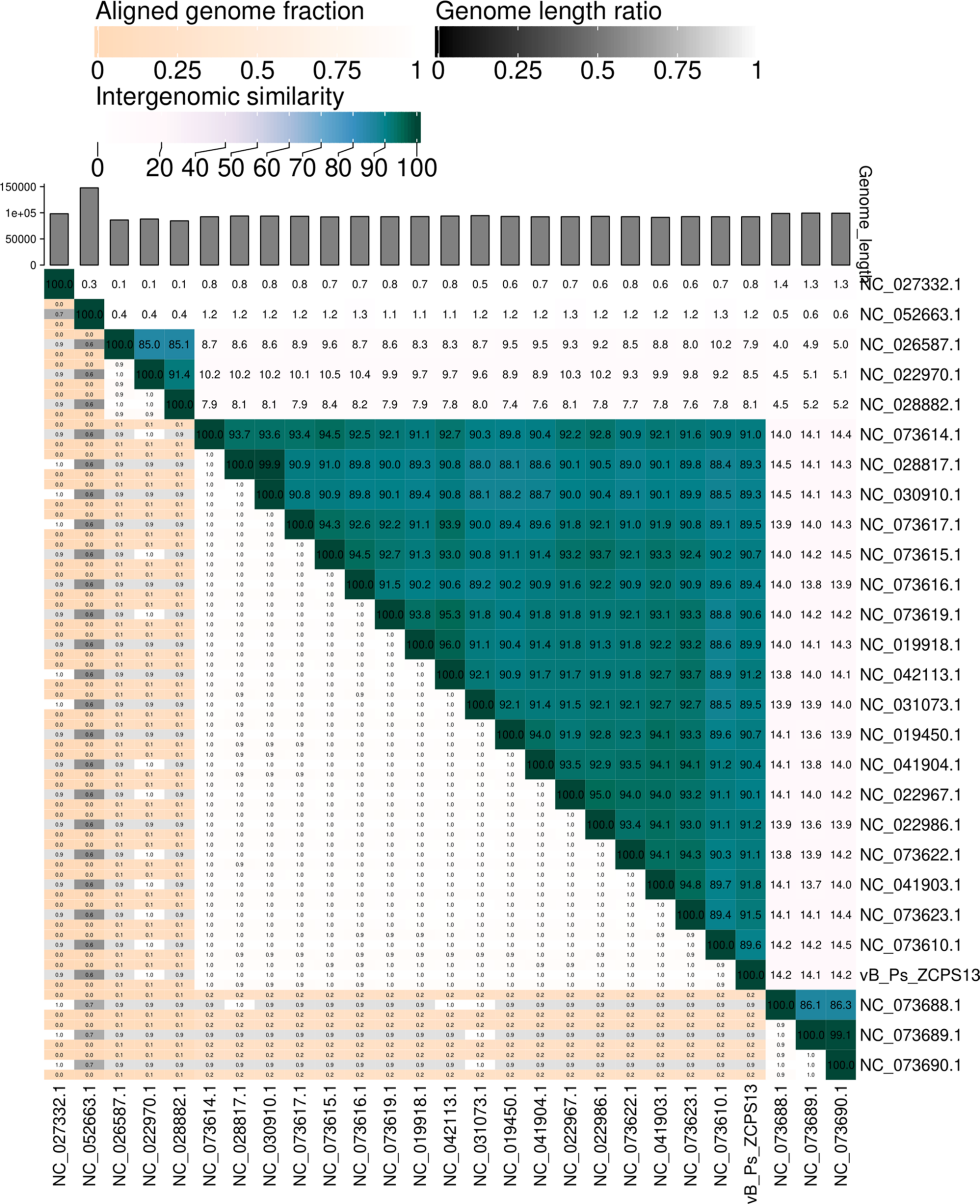
Phylogenetic analysis of phage vB_Ps_ZCPS13. (**a**) VIRIDIC heatmap for the intergenomic similarity between phage vB_Ps_ZCPS13 and closely related phages from ViPTree. (**b**) VIRIDIC heatmap for the intergenomic similarity between phage vB_Ps_ZCPS13 and all *Pakpunavirus* listed in the ICTV database.The alignment capacity is determined on the basis of three factors: intergenomic similarities (indicated by hues from green to blue), the percentage of aligned genome sequence in a pair (represented by shades of pink), and their length ratio (depicted by shades of black)

Proteome-based phylogeny was used to compare the genome of phage vB_Ps_ZCPS13 to 1,406 related phages using VipTree. Phage vB_Ps_ZCPS13 and the closely related phages bacterial hosts were found to belong to the *Pseudomonadota* phylum (Fig. 8a). Phage vB_Ps_ZCPS13 and 40 phages with the highest VipTree *S*_*G*_ were selected to construct a rectangular proteomic tree (Fig. 8b). The output of ViPtree was used to identify phages with the highest tBLASTx scores (*S*_*G*_) and outgroup phages with the lowest (*S*_*G*_) scores, which were used as input for the virus intergenomic distance calculator (VIRIDIC). The results of these intergenomic similarity comparisons were used to shortlist phages, and the analysis results were visually represented as a heatmap (Fig. 9a). VIRIDIC clustered phage vB_Ps_ZCPS13 along with 26 shortlisted phages into 23 species clusters. At the genus level, VIRIDIC clustered the phages into five different genera. The further analysis by VIRIDIC using all established species of *Pakpunavirus* listed in the ICTV database (Fig. 9b), confirmed that vB_Ps_ZCPS13 does not included in any known *Pakpunavirus* species, supporting its classification as a novel species within the genus.

**Fig. 10:**
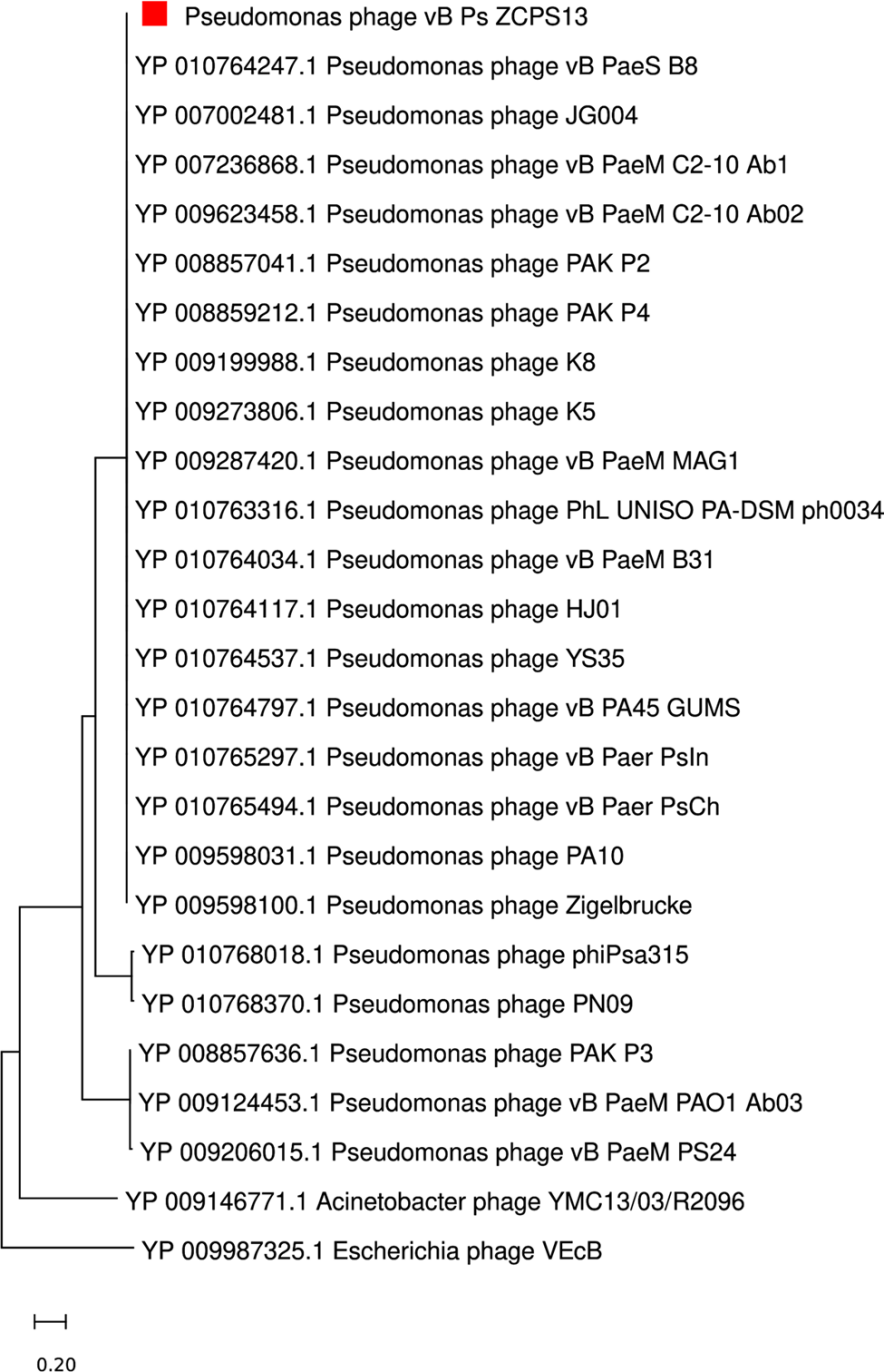
Phylogenetic tree based on a conserved viral signature protein (i.e., terminase large subunit) between phage vB_Ps_ZCPS13 and other phages. The tree was constructed by MEGA11

CoreGenes5 detected eleven homologous genes in vB_Ps_ZCPS13 and its 25 top-matched phages on BLASTn, which included the genes encoding terminase large subunit, tail sheath, endonuclease, and major head proteins. Accordingly, phylogenetic analysis based on terminase large subunit as a conserved signature protein was constructed by MEGA11, using CLUSTAL-W aligner and the best maximum likelihood fit model (Fig. 10) These results strongly suggest that phage vB_Ps_ZCPS13 is considered a *Pakpunavirus* in *Caudoviricetes.*

### Antibiofilm activity of phage vB_Ps_ZCPS13

**Fig. 11:**
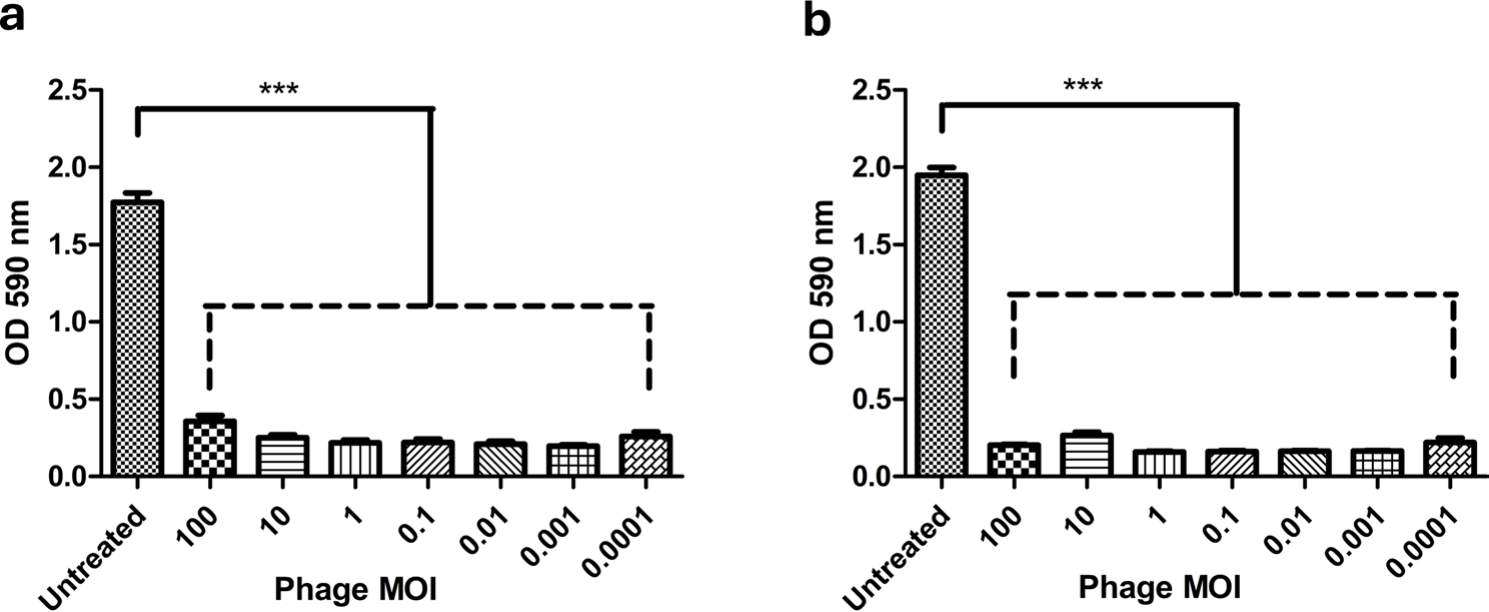
Antibiofilm activity of phage vB_Ps_ZCPS13. (**a**) Biofilm formation inhibition at different MOIs. (**b**) Disruption of pre-formed mature biofilm at different MOIs. Statistically significant difference marked by asterisks, where *** indicates *P* < 0.0001

Phage vB_Ps_ZCPS13 demonstrated significant inhibition of biofilm formation at all tested MOIs (*P* < 0.0001) compared to untreated cultures after 48 h of incubation (Fig. 11a). When exposed to a 48-hour biofilm, phage vB_Ps_ZCPS13 effectively disrupted the biofilm across all tested MOIs. After 24 h, the optical densities of the treated cultures were significantly (*P* < 0.0001) lower than those of untreated mature biofilm cultures (Fig. 11b).

### Antibiofilm activity of phage vB_Ps_ZCPS13 on urinary catheter surfaces

**Fig. 12:**
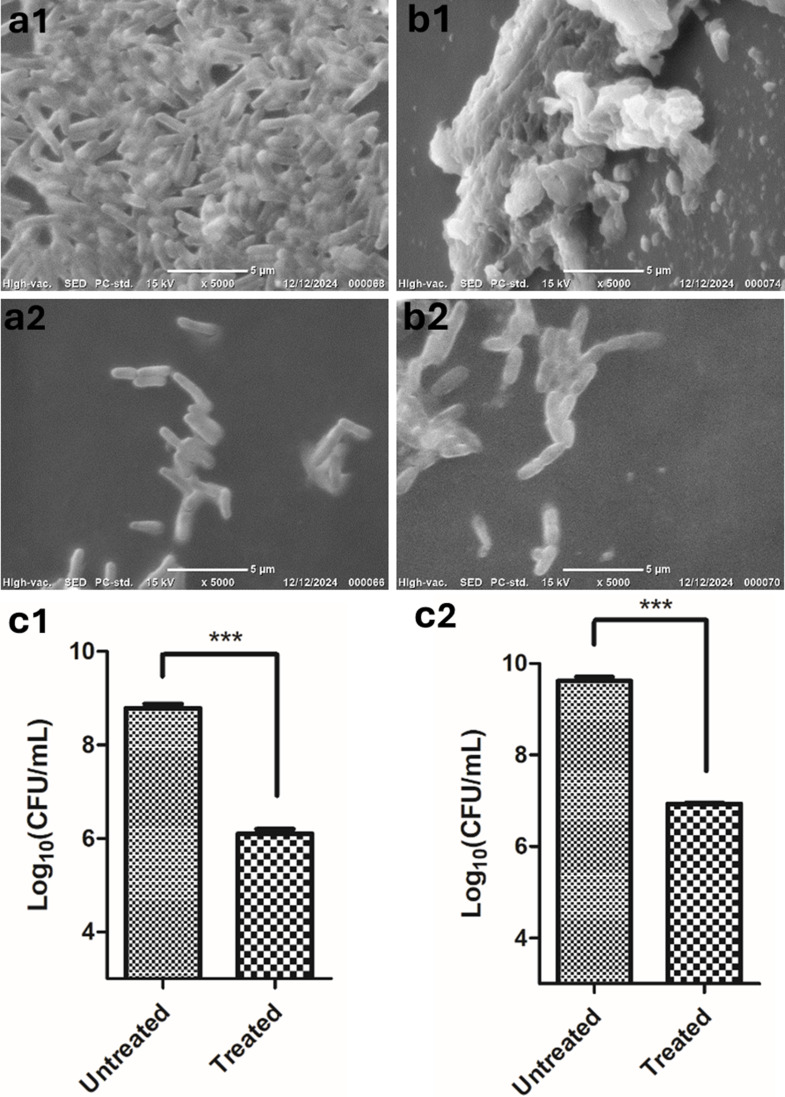
Representative SEM images of silicone urinary catheter surfaces treated with phage vB_Ps_ZCPS13. (a1 and b1) Untreated control for biofilm inhibition and biofilm clearance, respectively, (a2 and b2) phage-treated after 48 h for biofilm inhibition and biofilm clearance, respectively at magnification of 5000x. The relative number of CFU of *P. aeruginosa* biofilms formed in treated and untreated catheters for biofilm inhibition (c1) and biofilm clearance (c2). Statistically significant difference marked by asterisks, where *** indicates *P* < 0.0001

As observed through BT-SEM, the silicone urinary catheter slices confirmed the successful formation of a 48-hour biofilm (Fig. 12a1). In contrast, the phage vB_Ps_ZCPS13 demonstrated its ability to inhibit *P. aeruginosa* biofilm formation (Fig. 12a2). Similarly, BT-SEM confirmed the successful formation of a 72-hour biofilm (Fig. 12b1), while the phage vB_Ps_ZCPS13 exhibited degradation and a reduction in the *P. aeruginosa* population (Fig. 12b2). In the quantitative analysis, the CFU count decreased by approximately 3 log_10_ CFU/mL (*P* < 0.0001) during both biofilm inhibition (Fig. 12c1) and biofilm clearance (Fig. 12c2).

### Effect of phage vB_Ps_ZCPS13 on the viability of human cells

**Fig. 13:**
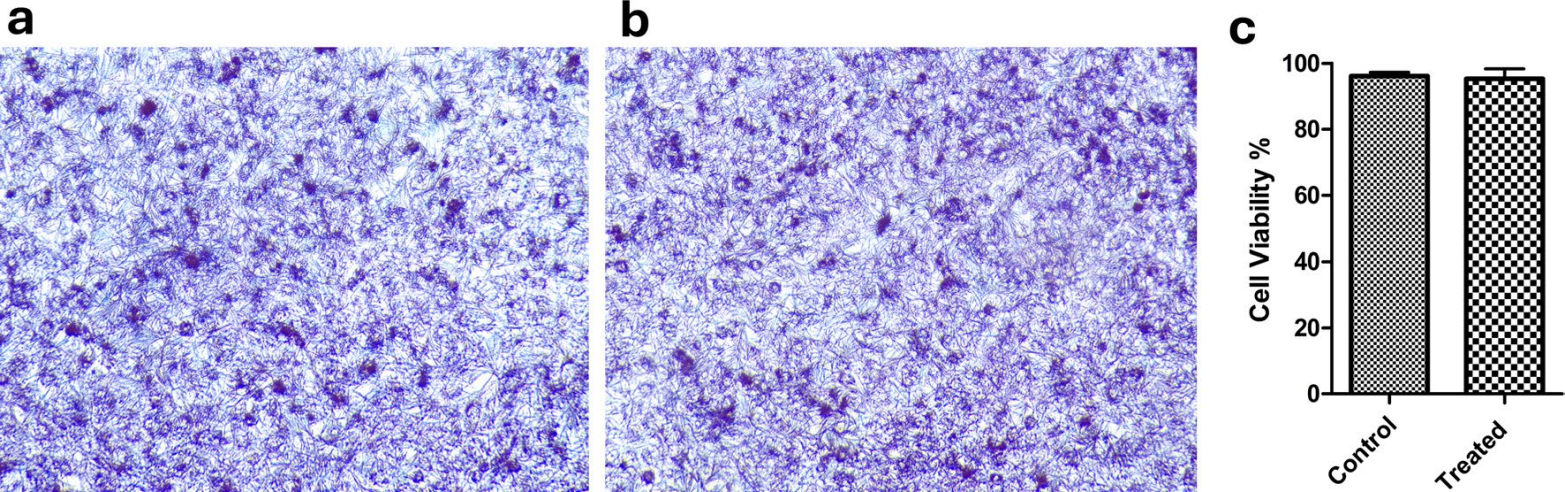
Phage vB_Ps_ZCPS13 cytotoxicity against normal human skin fibroblast (HSF) cell lines, as assessed by the MTT assay. (**a**) normal HSF cell lines, (**b**) treated HSF cell lines, (**c**) percentage of cell viability for both control and treated HSF cell lines

The cytotoxicity of phage vB_Ps_ZCPS13 revealed no significant reduction in HSF cell viability after 24 h of exposure to a high phage concentration (10¹⁰ PFU/mL), indicating that the phage does not exhibit any cytotoxic effects (Fig. 13).

## Discussion

The rise and spread of antimicrobial resistance (AMR) in *P. aeruginosa*, which is associated with hospital-acquired infections and urinary tract infections (UTIs), is a major global health concern. This is due to *P. aeruginosa*’s inherent resistance mechanisms, and its ability to form biofilms, making traditional antibiotics useless, particularly in catheter-associated urinary tract infections (CAUTIs) [52]. Phage therapy uses bacteriophages to kill pathogenic bacteria, and it is appealing due to its antibacterial activity and ability to break down biofilms. In this study, we isolated and characterized a virulent phage (vB_Ps_ZCPS13) against PDR *P. aeruginosa* and examined its prospects in the treatment of UTIs and CAUTIs.

In our study, antimicrobial susceptibility testing is particularly revealing a significant prevalence of antibiotic multidrug resistance across various antibiotic classes. The complete ineffectiveness of piperacillin (100%) against bacterial isolates, as well as the high ineffectiveness of the remaining antibiotics including cefepime (80%), gentamicin, tobramycin, and ciprofloxacin (75%), ticarcillin-clavulanate and amikacin (70%), ceftazidime (65%) piperacillin-tazobactam (60%), and imipenem (55%), demonstrates the widespread ineffectiveness of beta-lactams, aminoglycosides, and fluoroquinolones. These findings are consistent with global antimicrobial resistance (AMR) patterns, as numerous antimicrobial-resistant organisms, including *P. aeruginosa*, are widely distributed worldwide [53]. The Ps13 isolate was deliberately chosen for the current study because its PDR represents the prototypical severe form of the resistant phenotype that can be used to assess phage efficiency.

Phage vB_Ps_ZCPS13 has a wide host range, lysing 93% of the tested *P. aeruginosa*. Such a wide host range is necessary to resolve the issue of diversity in bacterial populations, especially within clinical facilities where *P. aeruginosa* has varying genetics and resistance profiles [54]. This finding correlates with the findings of Fei et al. and Shi et al. [55, 56]., who identified phage HZ2201 and phage_Pae01 with wide host ranges of 78.38% and 83.6%, respectively, against MDR *P. aeruginosa*.

The EOP values produced by four isolates were equal to one, indicating that these hosts were very permissive to phage replication, suggesting effective adsorption and intracellular replication. The other isolates that produced EOP values below one could either give rise to partial resistance or change in the availability of receptors [57, 58]. Such variability or heterogeneity of EOP values has been reported previously, underlying the host-dependent phage replication emphasized before [59, 60].

Phage vB_Ps_ZCPS13 infection against the bacterial host (Ps13) is suggested to be sustained for an extended period because of the low bacteriophage insensitive mutants (BIMs) frequency value, which is 0.001263 ± 0.000893. Such a low frequency of onset of resistance particularly emerges as encouraging because it implies that the phage can retain its lytic activity over a long period without considerable host of adaptation [61].

The ability of phage vB_Ps_ZCPS13 to preserve its integrity and infectivity after exposure to different conditions of temperature, pH, and UV light proves that phages can withstand a wide range of environmental conditions. Phage vB_Ps_ZCPS13 can remain active at 60 °C demonstrating thermal stability, which is rational with other studies on phages Pf-10, vB_PseuP-SA22, and JJ01 with similar temperatures stability [62-64]. However, a dramatic decrease of the titer at 70 °C and total inactivation at 75–80 °C suggests that there is a limit beyond which the phage undergoes structural damage, possibly from denaturation of critical proteins, also known as disintegration [65, 66]. This indicates that phage vB_Ps_ZCPS13 is favorable for use in areas with mild temperature variations.

Phage vB_Ps_ZCPS13, functioning within a broad range of pH (3.0–11.0), also demonstrates its robustness, making it convenient for use in various physiological and environmental conditions. This is useful for targeting infections at pH-variable sites like the urinary tract. Other phages such as phage vB_PsS_LDT325 specific to *P. aeruginosa*, have similar pH stability ranges, implying that a slight alkaline or acidic pH range is not sufficient to inactivate the phage [67]. Nevertheless, total inactivation of the phage at pH 2.0 and 12.0 is consistent with other observations that severe pH conditions result in permanent disruption of the phage capsid or its DNA, thus limiting the phage to highly alkaline or acidic conditions [68, 69].

Our study also demonstrated that phage vB_Ps_ZCPS13 becomes inactive with gradual intensity when subjected to UV exposure, with complete inactivation observed at 45 min, emphasizing the weak point of the phages when exposed to UV radiation. These findings are in line with the findings of a previous study related to three, phages PP1, PP2, and PP7, that were inactivated after 30 min of exposure to UV light [70].

The replication dynamics of phage vB_Ps_ZCPS13 demonstrated effective antibacterial activity across all tested MOIs, significantly reducing *P. aeruginosa* colony-forming units (CFUs/mL) and highlighting its potential as a promising antibacterial agent. Notably, the phage exhibited the highest antibacterial activity at a low MOI (0.1), as evidenced by a significant reduction in culturable bacteria within 300 min of the experiment. These results support the argument that lower MOIs favor effective virus replication, leading to sustained inhibition of the bacteria, as observed with the *Campylobacter* bacteriophage CP8, because the available bacteria are less saturated with excess viruses, thus improving the replication dynamics [71].

At MOI 10, it was observed that there was rapid control of bacterial growth. However, the bacteria also started regrowing after 120 min, which was quicker than during MOI 1 and 0.1. Most likely, this is a result of high MOIs, which restrict the number of susceptible bacterial hosts, thus limiting additional phage replication and resulting in the recovery of bacterial growth [72]. The higher replication of phage vB_Ps_ZCPS13 with MOI 0.1 confirms the “replication-first” hypothesis since lower initial titers of phage allow for more multiplication in a culture of susceptible hosts, thus amplifying the phage production [73, 74]. The emergence of phage insensitive mutants (PIM) or bacterial regrowth after near-complete lysis poses a challenge for phage therapy. However, previous studies have highlighted the importance of using phage cocktails or combining phages with antibacterials as adjunct therapies to reduce the risk of bacterial recovery and resistance development [73, 75]. The low frequency of BIMs observed in an earlier experiment with phage vB_Ps_ZCPS13 supports its utility as a monotherapy; however, combining it with other antimicrobials could further enhance its efficacy and reduce the risk of resistance.

Phage vB_Ps_ZCPS13 is suggested to be a member of the *Caudoviricetes* class, specifically a member of the *Pakpunavirus* genus, which is consistent with its characteristics of lytic and therapeutic activities [76]. The presence of 188 open reading frames (ORFs), which play central roles in replication, assembly, lysis, and infection, suggests the phage’s possibilities of being an alternative for antibiotics, especially against a highly resistant strain of *P. aeruginosa*. The presence of 12 tRNA genes in the vB_Ps_ZCPS13 genome further confirms the adaptability of this phage for concurrent multiplication inside the bacterial host and its ability to infect a broad range of hosts [77].

The functional annotation demonstrated that tail fiber proteins had high predictive confidence for depolymerase activity. In the presence of phages, bacterial exopolysaccharides are degraded by depolymerase to allow phage penetration through biofilms, a vital defense mechanism posed by *Pseudomonas* [75]. In addition, high-confidence detection of possible holin, a key protein for bacteriolytic action, was identified during transmembrane domain analysis, enhancing the lytic action of the phage. Moreover, the absence of temperate lifestyle genes, determinants of antimicrobial resistance or virulence factors, which were predicated by BACPHLIP and PhageLeads, demonstrated that this phage is safe for therapeutic application and factors set for phage therapy [78].

Comparative genomic analysis showed that phage vB_Ps_ZCPS13 is closely related to vB_Paer_PsCh (accession number NC_073623) [79] and PA10 (accession number NC_041903) [80, 81], sharing 91.5% and 91.8% genomic similarity, respectively. Among these closely related phages, vB_Ps_ZCPS13 exhibits superior performance, with the broadest host range (93%) and robust environmental stability, maintaining viability across a wide pH range [3–11] and temperatures up to 60 °C. In contrast, vB_Paer_PsCh displays a limited host range (23%) and reduced stability outside pH 5–9, while PA10 loses viability at pH 8 and temperatures above 50 °C. These traits highlight vB_Ps_ZCPS13 as the most promising candidate for therapeutic development due to its broad host spectrum and strong environmental tolerance.

The proteome-based analysis of phylogenetic relationships using VipTree and VIRIDIC contributed to the details required for the evolutionary development of phage vB_Ps_ZCPS13. It has been demonstrated that this phage clustered with other species of the *Pakpunavirus* genus while having distinct intergenomic distances with the closely related phages, indicating evolution with some degree of independence. An evolutionary marker such as conserved protein (terminase large subunit) was used to create a phylogenetic tree. This verified its belonging to the *Pakpunavirus*. Reflecting modern approaches in phage taxonomy and the significance of the importance of conserved proteins in proper classification, the study integrates protein and genome analysis [82–84].

The intergenomic-similarity clustering analysis done by VIRIDIC, as illustrated in which grouped phage vB_Ps_ZCPS13 into 23 species clusters belonging to five genera, also confirms the genetic diversity in vB_Ps_ZCPS13 lineage. The presence of 11 homologous genes with the best-matched phages, including the genes for important structural proteins, indicates functional conservation amongst these related phages. These findings agree with those of Andrade-Martínez et al. and Turner et al. [85, 86]., who highlighted that structural and enzymatic proteins which have been conserved are characteristic features of phage classification.

*P. aeruginosa* biofilm-associated strains are reported to have increased resistance mechanisms because of the biofilm, which serves as a protective matrix [87]. The ability of phage vB_Ps_ZCPS13 to prevent biofilm development and to break up biofilms that have already been formed, suggesting the potential as a treatment to prevent this type of biofilm-forming associated infection. The significant reduction *(P* < 0.0001) of biofilm formation for all tested MOI concentrations demonstrated that phage vB_Ps_ZCPS13 can selectively target and disrupt the early phases of biofilm formation. This finding is related to other investigations that have shown that bacterial viruses can interact with the first steps of biofilm formation, such as interfering with the initial adhesion of bacteria or with the production of enzymes that act on the extracellular matrix of biofilm [88]. Possible mechanisms of phage vB_Ps_ZCPS13 induced biofilms suppression are likely to occur when phage proteins, especially those present in tail fibers, bound to bacterial surface receptors, thus blocking bacterial attachment and biofilm formation [89]. Together with other evidence, this supports the hypothesis of vB_Ps_ZCPS13 in controlling *P. aeruginosa* biofilm infections.

The disruption of previously formed biofilms by phage of vB_Ps_ZCPS13 observed at all concentrations after 24 h of treatment denotes its lytic activity against established biofilms. Compared to control, the significant decrease in optical density (OD) in treated cultures suggests phage vB_Ps_ZCPS13 could enter biofilm matrices and then degrade them. Some investigations indicated that biofilm-degrading phages, including that described above, often carry depolymerases or other polysaccharide-degrading enzymes that enable the phage to respond to the bacterial cells in the biofilm [90, 91]. Phages PSP2, PSP3, and PSP30 infecting *P. aeruginosa*, for example, have been seen to contain certain depolymerases that contribute to their ability to colonize and infect biofilm structures [92]. Moreover, using bacterial enumeration and SEM imaging for biofilms on urinary catheter surfaces, the phage treatment demonstrated significant biofilm inhibition and degradation at MOI 1, resulting in a remarkable 3 log₁₀ CFU/mL reduction in bacterial counts (*P* < 0.0001). These results go along with those from earlier studies. For instance, Lehman & Donlan reported similar success in eradicating *P. aeruginosa* biofilms with treatment by bacteriophage cocktail including ΦPaer4, ΦPaer14, M4, ΦE2005-A, and ΦE2005-C considering a 4 log10 CFU/cm² reduction in biofilm count [93]. Additionally, Fu et al.., observed that bacterial biofilm density on urinary catheters was efficiently inhibited by M4 phage treatment, and that the pretreatment reduced the value by 2.84 log10 CFU/cm² [94].

Cytotoxicity was evaluated in this study by investigating the effects of bacteriophage vB_Ps_ZCPS13 on human skin fibroblasts (HSF) using an MTT assay. Despite high concentrations of phage (10^10^ PFU/mL) being used for 24 h, there was no substantial increase in the percentage of dead cells demonstrating that vB_Ps_ZCPS13 is safe in HSF cultures. This indicates that phage vB_Ps_ZCPS13 does not cause significant cytotoxicity to human cells, thus making it suitable for further use for therapeutic purposes in the treatment of infections without causing harm to tissues. These results are in accordance with several other works that investigated the cytotoxicity of bacteriophages and concluded that many therapeutic bacteriophages have exhibited low toxicity levels on human cells. For example, at therapeutic doses, the *P. aeruginosa* phages Motto and VbɸPA-1 were not cytotoxic to human cell lines, supporting the continued use of phage therapy as a possible alternative for antibiotics in the treatment of bacterial infections [95, 96].

One limitation of this study is the UV stability of phage vB_Ps_ZCPS13. While it is well-established that such sensitivity arises from DNA damage due to the UV-induced formation of pyrimidine dimers or cleavage of capsid proteins [97], such findings demonstrate the need to protect phage formulations from UV light during storage or use in an outdoor environment. Genetic engineering, encapsulating, or embedding into UV-protective matrices might help improve its stability when keeping it in a UV-lighted environment is unavoidable.

## Conclusions

In summary, the findings of this study indicate that phage vB_Ps_ZCPS13 is a prospective therapeutic agent for pan-drug-resistant *P. aeruginosa*, especially in the context of biofilm-associated infections. The phage exhibited extensive lytic activity against 93% of the clinical isolates tested, including the PDR Ps13 strain, demonstrating excellent efficacy in inhibiting and destroying mature biofilms. The study demonstrated potent in vitro antibacterial activities over a wide range of multiplicities of infection with maximal efficacy at lower MOIs. Furthermore, the phage exhibited good stability under various conditions, maintaining its effectiveness even when exposed to different temperatures and pH values. These findings suggest that vB_Ps_ZCPS13 could be safely utilized as an alternative or adjuvant to antibiotics in the treatment of MDR *P. aeruginosa* infections, although further in vivo studies are needed.

## Data Availability

The dataset presented in this study can be found in NCBI GenBank. The annotated genome sequence of the phage vB_Ps_ZCPS13 was deposited under the accession number PQ656807 with taxonomy ID 3385019.

## Abbreviations

UTIs: Urinary Tract Infections
PDR: Pan-Drug Resistance
PFGE: Pulsed-Field Gel Electrophoresis
TEM: Transmission Electron Microscopy
SEM: Scanning Electron Microscopy
HSF: Human Skin Fibroblast
MAR: Multiple Antibiotic Resistance
EOP: Efficiency of Plating
BIM: Bacteriophage-Insensitive Mutants
CAUTIs: Catheter-Associated Urinary Tract Infections
WHO: World Health Organization
P. aeruginosa: Pseudomonas aeruginosa
MDR: Multidrug-Resistant
AMR: Antimicrobial Resistance
CMP: Center for Microbiology and Phage Therapy
CLSI: Clinical and Laboratory Standards Institute
PFU: Plaque-Forming Units
CFU: Colony-Forming Units
RASTtk: Rapid Annotation using the Subsystem Technology Toolkit
BACPHLIP: BACterioPHage LIfestyle Predictor
TMDs: Transmembrane Domains
PhageDPO: Phage Depolymerase Finder
VIRIDIC: Virus Intergenomic Distance Calculator
TerL: Terminase Large Subunit
DMEM: Dulbecco’s Modified Eagle’s Medium
OD: Optical Density
SD: Standard Deviation
ICTV: International Committee on Taxonomy of Viruses
CDS: Coding Sequences
PIM: Phage Insensitive Mutants
ORFs: Open Reading Frames
